# Mid-term follow-up results after implementing a new strategy for the diagnosis and management of periprosthetic joint infections

**DOI:** 10.1186/s12879-021-06407-x

**Published:** 2021-08-12

**Authors:** Rares Mircea Birlutiu, Manuela Mihalache, Patricia Mihalache, Razvan Silviu Cismasiu, Victoria Birlutiu

**Affiliations:** 1 Lucian Blaga University of Sibiu, Faculty of Medicine Sibiu, Str. Lucian Blaga, Nr. 2A, 550169 Sibiu, Romania; 2 Lucian Blaga University of Sibiu, Bd-ul. Victoriei, Nr.10, 550024 Sibiu, Romania; 3 Carol Davila University of Medicine and Pharmacy, Bucharest, Romania; 4 FOISOR Clinical Hospital of Orthopedics, Traumatology, and Osteoarticular TB Bucharest, Str. Dionisie Lupu nr. 37, Sector 2, 020021 Bucharest, Romania; 5 Academic Emergency Hospital Sibiu - Infectious Diseases Clinic, B-dul Corneliu Coposu, Nr.2-4, 550245 Sibiu, Romania

**Keywords:** Periprosthetic joint infection, Sonication, Surgical management, Biofilm, Mid-term follow-up results

## Abstract

**Background:**

Periprosthetic joint infections (PJIs) represent one of the most serious complications associated with joint replacement surgeries, a complication also of modern orthopedic surgery despite the efforts that occurred in this field. Frequently PJIs lead to prolonged morbidity, increased costs and mortality.

**Methods:**

We are conducting a single-center observational cohort ongoing study in the Academic Emergency Hospital Sibiu, Romania, study in which sonication of the retrieved and as a rapid method of bacteria detection, molecular identification of bacteria by 16S rRNA beacon-based fluorescent in situ hybridization (bbFISH) are used.

**Results:**

A total of 61 patients were enrolled in this study. The diagnosis of aseptic loosening was established in 30 cases (49.1%) and the diagnosis of periprosthetic joint infection was established at 31 patients (50.8%). The mean follow-up period in the subgroup of patients diagnosed with periprosthetic joint infections was 36.06 ± 12.59 months (range: 1–54). The 25-months Kaplan-Meier survival rate as the end point, as a consequence of the period of enrollment and a different follow-up period for each type of surgical procedure, was 75% after debridement and implant retention, 91.7% after one-stage exchange, 92.3% after two-stage exchange, and 100% after three-stage exchange. There were no significant differences in survival percentage.

**Conclusions:**

Our study has good results similar to previously published data. We cannot recommend one strategy of managing prosthetic joint infections over the other. Definitely, there is a need for prospective randomized controlled trials.

## Background

Although Anthony van Leeuwenhoek (1632–1723), observes and describes the bacterial biofilm by using a rudimentary microscope analyzing specimens of tissue from his oral cavity, on which he highlights the presence of aggregates of microbes in the “scurf of the teeth” and from “particles scraped off his tongue” between 1683 and 1708, accelerated research on the study of the biofilm only began to make important discoveries in the last 20–25 years [1]. Unofficially the total annual cost of the management of biofilm-related infections in the United States is around $94 billion and more than half a million deaths are associated with this economic burden. A United States Centers for Disease Control and Prevention report from 2007 is estimated that in the US alone 1.7 million hospital-acquired infections annually are biofilm-related infections, requiring an additional $11 billion in healthcare costs for the management of these patients [2, 3]. Dental caries and periodontitis are the most prevalent biofilm-related infections requiring in the United States alone $105 billion on dental care [4]. The spectrum of biofilm-related infections is comprehensive, from catheter-associated urinary tract infections that are the most common device-related biofilm infections to central line-related blood stream infections, periprosthetic joint infections, biofilm-related infection associated with the presence of fixed braces [5, 6] and ventilator-associated pneumonia are also of important concern [7]. Periprosthetic joint infections and catheter-associated urinary tract infections are, probably, the two most important biofilm-related foreign body infections, associating a great economic impact in health care [2]. The ESKAPE group of pathogen contains highly virulent and antibiotic resistant bacterial pathogens, most of these strains being biofilm-producers. Strains that pose a significant interest in studying the possible relationship between the biofilm-producer phenotype and their MDR status. Several studies have been published and provide data on this relationship, data from both MDR and wild-type strains (eg, *Escherichia coli* or MSSA/MRSA). The conclusions of these studies currently available data are frequently controversial [8, 9].

Periprosthetic joint infections (PJIs) are one of the most serious complications associated with joint replacement surgeries [10], a complication also of the modern orthopedic surgery despite the efforts that occurred in this field. Frequently PJIs lead to prolonged morbidity, increased costs and mortality [11, 12]. PJIs continue to develop in increasing numbers despite all the international efforts, the rate of PJI is estimated to range between 0.5 and 2.4% after primary total hip and knee arthroplasties, and up to 20% in cases of revision surgeries [13, 14]. An accurate diagnosis of PJIs is crucial for treatment outcome especially having in mind the fact that the management of aseptic loosening is different from the one of PJIs. The management of PJIs remains a difficult one.

In this setting, the beginning of September 2016 was an important key point in time for the Academic Emergency Hospital Sibiu, Romania when a new strategy for the diagnosis and management of the periprosthetic joint infections was adopted and implemented, a strategy that uses sonication of the retrieved and as a rapid method of bacteria detection, a molecular identification of bacteria by 16S rRNA bbFISH (beacon-based fluorescent in situ hybridization) technology using a bbFISH kit (hemoFISH Masterpanel, miacom diagnostics GmbH Düsseldorf, Germany).

## Materials and methods

### Study design

We are conducting a single-center observational cohort ongoing study in the Academic Emergency Hospital Sibiu, Romania, a county hospital with 1054 beds. Before patient inclusion in the study, the study protocol was reviewed and an approval from the institutional review board was received. A standardized diagnostic system was used for all patients that underwent a surgical intervention of revision of a joint prosthesis, to assess the implant failure. Our new implemented diagnostic and management strategy included a standardized sampling of at least 4 intraoperative tissue samples (1 of the samples being used for the histopathological examination (periperiprosthetic membrane) and the other are sent to the microbiological laboratory for bacterial cultures), sonication of retrieved orthopedic implants (periprosthetic components or polymethylmethacrylate (PMMA) spacer) and harvesting of the sonication fluid, a complex analysis of the synovial fluid, and also a state of the art assessment of the sonication fluid using a bbFISH kit (hemoFISH Masterpanel, Miacom diagnostics GmbH Düsseldorf, Germany) as a rapid method of bacteria detection (an assay that combines the well-known classical FISH technology with the usage of fluorescently labelled DNA-molecular beacons as probes). All biological sample that required cultures were inoculated and incubated aerobically, anaerobically and in high concentration of CO_2_ (GENbag-GENbox Atmospheric generators bioM rieux, Marcy-l’Étoile, France) at 37 °C. Isolated bacteria are identified using the VITEK 2 Compact analyzer (bioMérieux, Marcy-l’Étoile, France). The minimum inhibitory concentrations are assessed according to the European Committee on Antimicrobial Susceptibility Testing breakpoints. Synovial fluid was analyzed for cellularity, C-reactive protein levels, and leukocyte esterase. Also, a 14-day period of incubation was implemented. The full details of the implemented protocol were published in some of our previous articles [15, 16].

### Study population

We prospectively included in this study all consecutive patients aged over 18 years, that were hospitalized from September 2016 through January 2019, patients that underwent a joint arthroplasty revision surgery for any reason. Detailed information regarding the enrolled subjects was abstracted from the medical records. We evaluated the retrieved information for the following data: demographic characteristics; clinical, radiographic, laboratory, histopathological, and microbiological data; type of surgical management, and antimicrobial management. Complete data were available for all the participants in the study. The enrolled patients were followed until they develop a treatment failure, died, or were lost during the follow-up period. The follow-up period was extended until April 2021, with the study population being followed for a maximum period of 55 months. The data were statistically analyzed using IBM SPSS Statistics® version 26 software. The level of statistical significance was set at *p* < 0.05. Kaplan-Meier survival curves are widely used in clinical and fundamental research. The Kaplan-Meier estimator, or Kaplan-Meier estimation function, is used to estimate the survival function or survival rate. The visual representation of this function is usually called the Kaplan-Meier curve and shows the probability of an event (for example, survival) at a certain point in time. If the sample size is large enough, the curve should approach the true survival rate of the research population. These curves are usually generated to assess the survival rate of patients at different statistical intervals such as the 30 days survival rate. To generate Kaplan – Meier curves in the therapeutic management of patients diagnosed with periprosthetic joint infections, we used the Kaplan – Meier survival function of the IBM SPSS Statistics® version 26 software. Since no death events were recorded in this research, we defined the survival rate by the absence rate of infection and mortality by the recurrence rate of the infection.

### Study definitions and classification

Periprosthetic joint infection was defined using the criteria from the workgroup of the Musculoskeletal Infection Society published by Javad Parvizi et al. [17] We used the classification proposed by Zimmerli et al. to determine if there is an acute, late chronic, or acute late periprosthetic joint infection, a classification that defines the prosthetic joint infections as early (occurring within 3 months after surgery), delayed (3–24 months) and late (> 24 months) [18]. We also used a much simpler classification, a classification from the Pocket Guide to Diagnosis & Treatment of Periprosthetic Joint Infection (PJI) of the PRO-IMPLANT Foundation, Berlin, Germania (coordinated by N. Renz and A. Trampuz) a guide that is in line with national and international recommendations and that defines periprosthetic infections as acute or chronic (Perioperative/Hematogenous or per continuitatem).

## Results

A total of 61 patients were enrolled in this study during the analyzed period, representing a total number of 61 retrieved implants, eighter endoprosthesis (*n* = 58), or PMMA spacers (*n* = 3). The diagnosis of aseptic loosening of an endoprosthetic implant was established in 30 cases (49.1%) and the diagnosis of periprosthetic joint infection was established at 31 patients (50.8%). Thus, in 2016, 14 patients were enrolled (23%), 7 of them being diagnosed with aseptic loosening and the rest with periprosthetic joint infection. In 2017, 19 patients were enrolled (31.1%), 13 patients belonging to the study group with patients diagnosed with aseptic loosening, and 6 patients diagnosed with a periprosthetic joint infection. In 2018, 17 patients were enrolled (27.9%), 8 patients were diagnosed with aseptic loosening and 9 patients diagnosed with a periprosthetic joint infection, and in 2019 11 patients were enrolled (18%), 2 patients belonging to the study group with patients diagnosed with aseptic loosening and 9 patients diagnosed with a periprosthetic joint infection. There were no statistically significant differences between the 2 study groups in terms of the patient enrollment period (*p* = 0.690). Of the 30 retrieved implants from the 30 patients diagnosed with aseptic loosening of an endoprosthetic implant, 16 of them were hip and 14 knee implants, and in the group of patients diagnosed with a periprosthetic infection, 14 hip implants, 14 knee implants, and 3 PMMA preformed hip spacers. Regarding the PMMA spacers, the initial surgeries (the first stage of a two-stage revision surgery) were performed before the introduction of the proposed diagnostic strategy and no pathogen was isolated. Overall (*n* = 61) the mean age of the studied population was 67.62 ± 8.058 years (range: 44–83 years). In the subgroup of patients diagnosed with aseptic loosening, the mean age of the patients was 68.50 years (range, 44–83 years, standard deviation ±8.768). In the subgroup of patients diagnosed with a periprosthetic joint infection, the mean age of the patients was 68 ± 7.422 years (range: 49–83 years). Twenty-nine patients were male patients (47.5%) and 32 patients were living in rural areas in the entire study group. Twenty-two patients were female patients (73.3%) and 17 patients were living in rural areas (56.6%) in the subgroup of patients diagnosed with aseptic loosening and in the subgroup of patients diagnosed with a periprosthetic joint infection, 10 patients were female patients (32.2%) and 15 patients were living in rural areas (48.3%). There were no statistically significant differences between the 2 study groups related to age or background of the patients, *p* = 0.574, respectively *p* = 0.517. From the gender point of view, there were statistically significant differences between the 2 subgroups, *p* = 0.001.

Using the classification proposed by Zimmerli et al., we were able to group the 31 patients diagnosed with periprosthetic joint infection as follows: 9 patients diagnosed with early periprosthetic joint infection, 6 patients with delayed periprosthetic joint infection and 16 patients were diagnosed with a late periprosthetic joint infection.

Using the classification of the periprosthetic joint infections proposed in the Pocket Guide by the PRO-IMPLANT Foundation, 5 patients were diagnosed with an acute perioperative infection, 4 patients with acute hematogenous infection, and 22 patients with chronic prosthetic joint infection. The number of patients diagnosed with an acute periprosthetic joint infection in 2016 was 2, in 2017 1, in 2018 1, and 2 cases in 2019. Patients diagnosed with a hematogenous acute periprosthetic joint infection in 2016 were 0, in 2017 1, in 2018 0, and in 2019 2 cases. Regarding the patients diagnosed with a chronic periprosthetic joint infection in 2016, there were 0 cases, in 2017 4, in 2018 8, and 5 patients had been diagnosed in 2019.

We analyzed all 31 cases of periprosthetic joint infections from a therapeutic management point of view. Surgical management was associated with specific antibiotic therapy in all cases. None of the enrolled patients were managed with long-term suppressive antibiotic therapy (Table 1.)

**Table 1:** Distribution of the number and the type of surgeries by type of periprosthetic joint infections

Surgical intervention	Type of periprosthetic joint infection		
	Acute	Acute hematogenous	Chronic
**3SE - Three-stage exchange**	–	–	1
**TSE - Two-stage exchange**	1	–	12
**OSE - One-stage exchange**	2	1	9
**DAIR - Debridement and implant retention**	2	3	–

The implemented therapeutic strategy failed in two cases.

Patients were followed up and evaluated postoperatively until they developed treatment failure, died, or were lost during the follow-up period. During the study period from the available documents no patient died, and no patient was lost during study.

The mean follow-up period in patients diagnosed with aseptic loosening of an endoprosthetic implant was 42.73 ± 8.835 months (range: 26–54) with a standard error of 1.613 months, and a 95% Confidence Interval for Mean with a lower bound of 39.43 months and an upper bound of 46.03 months.

The mean follow-up period in the subgroup of patients diagnosed with periprosthetic joint infections was 36.06 ± 12.596 months (range: 1–54) with a standard error of 2.262 months, and a 95% Confidence Interval for Mean with a lower bound of 31.44 months and an upper bound of 40.68 months.

### Kaplan-Meier survival analysis of the study group (*n* = 61)

**Fig. 1:**
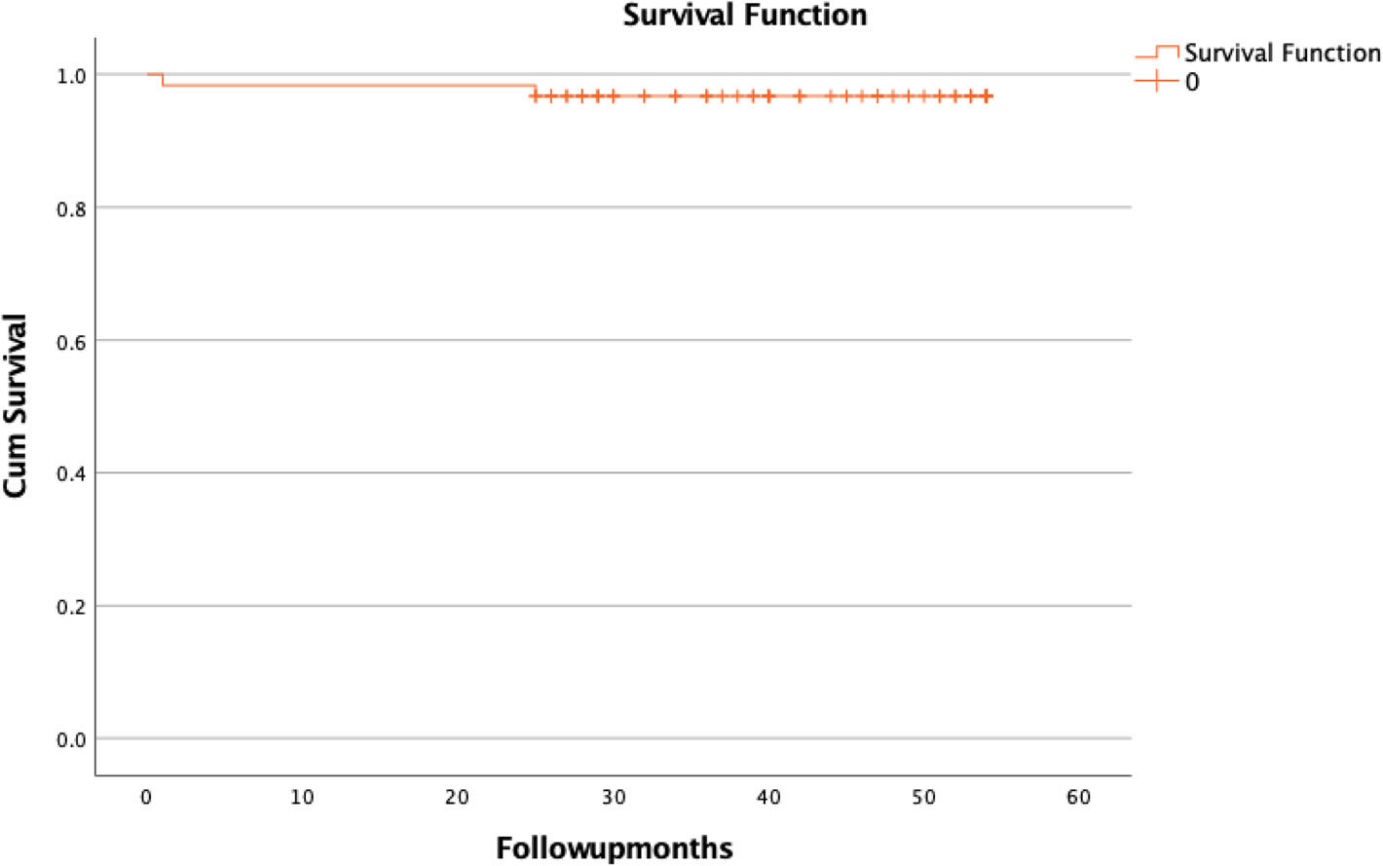
Kaplan-Meier survival plot of the study group (*n* = 61)

Analyzing the cumulative survival table, the cumulative probability of mortality at 30 days is 1.6%, at 25 months 3.3%, and at 54 months 3.3% or the cumulative probability of survival at 30 days is 98.4%, at 25 months 96.7%, and at 54 months 96.7%. (Fig. 1).

### Kaplan-Meier survival analysis on study subgroups

**Fig. 2:**
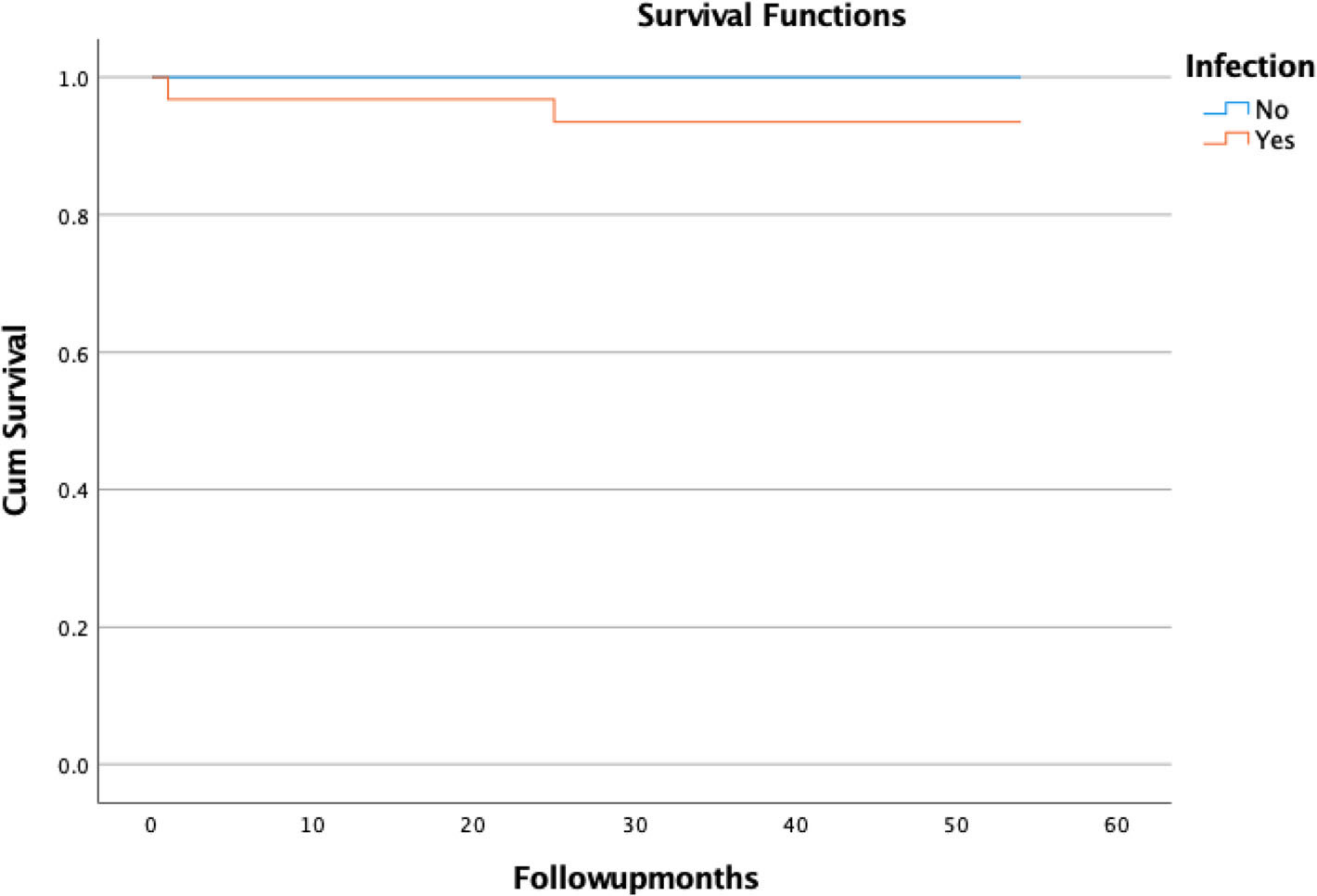
Kaplan-Meier survival plot of the study group by infection

We performed this analysis on the two study groups. Again, after assessing the cumulative survival table, the cumulative probability of mortality in patients diagnosed with aseptic loosening of the implant, at 12 months 0%, and at 54 months 0% or the cumulative probability of survival at 12 months 100%, and at 54 months 100%. Regarding the patients diagnosed with periprosthetic joint infection the cumulative probability of mortality at 1 month 3.2%, at 25 months 4.4%, and at 54 months 4.4% or the cumulative probability of survival at 1 month is 96.8%, at 25 months 93.5%, and at 54 months 93.5%. (Fig. 2).

As several surgical procedures have been used in the subgroup of patients diagnosed with a periprosthetic joint infection, an analysis according to them is also necessary.

### Kaplan-Meier survival analysis of the PJIs subgroups by type of surgical procedure

As previously mentioned, the therapeutic management implemented failed in two cases. In the first case, the failure was caused by a lack of compliance with the antibiotic regimen (the patient interrupted the ciprofloxacin therapy at home after 30 days), in the context of an immunocompromised patient that was on immunosuppressive therapy for a kidney transplant. As a method of treatment, a resection arthroplasty was performed – a Girdlestone procedure, being a case of failed treatment for a recurrent infection associated with a total hip arthroplasty. At 54 months of follow-up since the last surgery, there are no signs of reinfection. In the second case, a periprosthetic joint infection caused by a bacterial strain of *Enterococcus faecalis* (at 25 months after the revision surgery, the patient returns for pain-like complaints in the hip, associated with a fistula; a two-stage exchange procedure was performed). The patient is free of infection at the last fallow-up (Table 2.)

**Table 2:** Follow-up period by type of surgical management

Statistic		Surgery			
		Aseptic Loosening Intervention	DAIR	OSE	TSE
**Mean**		42.73	28.25	38.50	35.21
**95% Confidence Interval for Mean**	**Lower Bound**	39.43	26.25	32.39	26.13
	**Upper Bound**	46.03	30.25	44.61	44.30
**5% Trimmed Mean**		43.02	28.22	38.39	36.07
**Median**		43.00	28.00	39.00	29.50
**Variance**		78.064	1.583	92.455	247.566
**Std. Deviation**		8.835	1.258	9.615	15.734
**Minimum**		26	27	25	1
**Maximum**		54	30	54	54
**Range**		28	3	29	53

*Follow-up months is constant when Surgery = 3SE. Data has been omitted

**Fig. 3:**
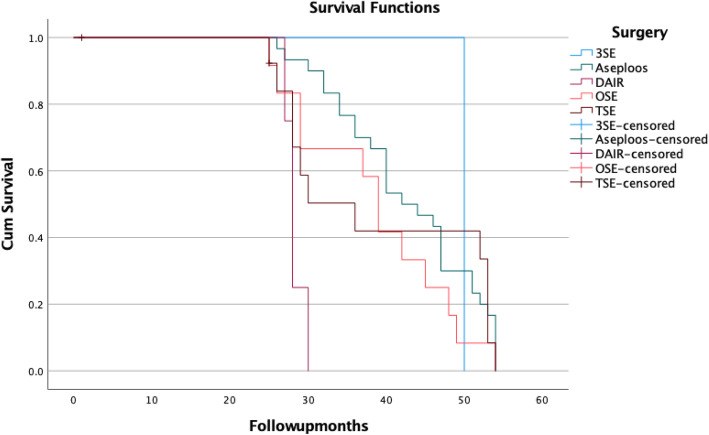
Kaplan-Meier survival plot of the study group by type of surgical procedure

For this analysis, we will report the data for a period of 25 months of follow-up (as a consequence of the period of enrollment and a different follow-up period for each type of surgical procedure), and in Fig. 3 is the survival function (estimation that is limited to the largest survival time) (Fig. 3, Table 3).

**Table 3:** Kaplan-Meier survival index by procedure, and date

Surgery	Time (months)	Kaplan-Meier Survival at the Time
**3SE**	12	100.00%
	25	100.00%
**TSE**	12	100.00%
	25	92.30%
**OSE**	12	100.00%
	25	91.70%
**DAIR**	12	100.00%
	25	75.00%
**Aseptic loosening intervention**	12	100.00%
	25	100.00%

## Discussion

The diagnosis and management of periprosthetic joint infections remain an issue. The adapted protocols of the management of biofilm-related infections and new diagnostic methods have improved the rate of eradication, without having a 100% certainty that the infections have been eradicated. Well-equipped treatment centers for diagnosis and multidisciplinary teams of surgeons-infectious disease specialists - clinical microbiologists are needed. Periprosthetic joint infections are the most feared complication associated with joint arthroplasty and require an early, rapid, and accurate diagnosis, that can lead to the implementation of an adapted therapeutic management strategy. PJIs are complications that are associated with increased length of hospitalization, frequent readmissions and surgeries, an increased cost of management, and increased morbidity and mortality [3, 14, 19].

During our follow-up period, based on the two cases of treatment failure associated with a two-stage exchange intervention, we can state that, in the case of this group of studied patients, the recurrence rate of a periprosthetic joint infection is 6.45%.

The 25-months Kaplan-Meier survival rate as the end point, as a consequence of the period of enrollment and a different follow-up period for each type of surgical procedure, was 75% after debridement and implant retention, 91.7% after one-stage exchange, 92.3% after two-stage exchange, and 100% after three-stage exchange. There were no significant differences in survival percentage. No significant difference in survival rate between patients that underwent a one-stage or two-stage exchange was also published in previous studies [20–26]. Success rates associated with a debridement and implant retention strategy published in the last 20-years are between 31 and 82% depending on the involved pathogen [27–34]. Buchholz et al. published in 1981 a large study reporting a success rate of 77% for a periprosthetic joint infection associated with hip arthroplasty [35]. Other studies reported success rates between 84 and 100% [36–39]. Two-stage exchange arthroplasty is generally an effective strategy for the management of periprosthetic joint infection, with success rates reported in the case of hip PJI between 87 and 100% [40–42] Studies that enrolled periprosthetic joint infection cases associated with total knee arthroplasty managed with a two-stage exchange protocol have reported success rates between 72 and 95% [43–48] Some systematic reviews have reported a higher success rate with two-stage exchange in the eradication of infection when analyzing knee periprosthetic joint infections [49, 50].

There is a constant debate between which is the standard for treating PJIs, some studies suggest that the two-stage exchange procedure is the standard, other studies claimed that two-stage exchange procedures are complex and lead to increased morbidity and a less favorable functional result, these studies advocate for a one-stage exchange strategy [51–54]. Analyzing the type of surgical procedure implemented in our study, depending on the type of periprosthetic joint infection, and the year of enrollment of the patients, differences were observed in terms of the adopted strategy. Differences that in our opinion, most likely, occurred with the increase of the confidence level in the diagnostic and management strategies implemented through this research and with the publication, in the literature, of the long-term results obtained following the use of the same strategies in different reference centers at international level.

Second-generation lipoglycopeptide antibiotics that are used to treat acute bacterial skin and skin structure infections wight be options for difficult to treat PJIs [55]. Nasir et al. evaluated the efficacy of antibiotic combinations by calculating the fractional inhibitory concentration index (FICI) using the minimum inhibitory concentration; an FICI ≤0.5 was interpreted as “synergistic”. An antibiotic combination was interpreted as “synergistic” when ≥2-log10 reduction in growth was observed in the colony forming units. The use of amoxicillin-clavulanic acid combinations with cephradine seems to be synergistic with XDR methicillin-resistant *S. aureus*, and the combination with ciprofloxacin is effective against Gram-negative bacteria [56]. A constant increase of the lack of susceptibility of the isolated strain from PJIs and also the lack of new and cost-efficient antibiotic management strategies may encourage the development of natural treatments, there are some studies that consider it useful to use biologically active phytocomplexes instead of antibiotic therapy, consequently reducing bacterial resistance to commonly used antibiotics or of Lavender essential oils (Lavanda sumian and Lavanda grosso) that significantly reduce the nitric oxide synthase activity in a cell model [57, 58]. Different nanomaterials in biomedicine have studied for their unique properties, silver nanoparticles being one of them due to their good antibacterial activity being and alternative option in wound and bone healing. The use of a silver-containing hydroxyapatite (Ag-HA) coating in combination with vancomycin has a suppressive effect in cases of biofilm associated infections (PJIs) with methicillin-resistant *Staphylococcus aureus*, might being a useful strategy of the prevention of methicillin-resistant *Staphylococcus aureus* -associated PJIs [59, 60].

Delayed and late infections are predominantly caused by low-virulent microorganisms like Coagulase-negative staphylococci and anaerobes (eg *Cutibacterium* species). The proportion of PJI caused by anaerobes varies from 3 up to 24%. An early recognition and management of these PJIs is important, as the mortality rate is increasing and having in mind the fact that many anaerobic species have developed resistance. The use of different bacteria culture media could increase the rate of diagnosis of PJIs caused by anaerobes. Lytic (BACTEC) and SN (BacT/ALERT) blood culture media increased the detection rate of *Cutibacterium acnes* [61–63].

The treatment strategy, in our opinion, should finally be selected by the surgeon, in association with the patient, and together with the input of the infectious disease specialist. Several authors and expert groups have suggested algorithms to help choose the right treatment strategy for each patient [18, 64–66].

Our study also had some limitations. First, the type of the study, a monocentric, observational, cohort study. Second, the small population of enrolled patients in the study, respectively the number of cases with periprosthetic joint infections included associated with a relatively short period of enrollment and follow-up. Third, the center where this study is conducted is not a dedicated center for the treatment of periprosthetic joint infections, but with the introduction of the new protocol and the dedicated team to manage these cases (orthopedic surgeon - infectious disease specialist - microbiologist), the results are encouraging. Larger studies are needed to confirm these results. However, our results are very promising.

## Conclusions

Our study has good results similar to previously published data. Because of our study limitations (small number of patients and a relatively short period of follow-up), we cannot recommend one strategy of managing prosthetic joint infections over the other. Definitely, there is a need for prospective randomized controlled trials comparing treatment strategies to validate our data.

## Data Availability

All data generated or analysed during this study are included in this published article and are available from the corresponding author on reasonable request.

## Abbreviations

PJI: Periprosthetic joint infections
rRNA: Ribosomal ribonucleic acid
bbFISH: beacon-based fluorescent in situ hybridization
PMMA: Polymethylmethacrylate
3SE: Three-stage exchange
TSE: Two-stage exchange
OSE: One-stage exchange
DAIR: Debridement and implant retention
